# Heat stress at the bicellular stage inhibits sperm cell development and transport into pollen tubes

**DOI:** 10.1093/plphys/kiae087

**Published:** 2024-02-15

**Authors:** Xingli Li, Astrid Bruckmann, Thomas Dresselhaus, Kevin Begcy

**Affiliations:** Department of Cell Biology and Plant Biochemistry, University of Regensburg, 93053 Regensburg, Germany; Department for Biochemistry I, Biochemistry Centre, University of Regensburg, 93053 Regensburg, Germany; Department of Cell Biology and Plant Biochemistry, University of Regensburg, 93053 Regensburg, Germany; Environmental Horticulture Department, University of Florida, Gainesville, FL32611, USA

## Abstract

For successful double fertilization in flowering plants (angiosperms), pollen tubes deliver 2 nonmotile sperm cells toward female gametes (egg and central cell, respectively). Heatwaves, especially during the reproduction period, threaten male gametophyte (pollen) development, resulting in severe yield losses. Using maize (*Zea mays*) as a crop and grass model system, we found strong seed set reduction when moderate heat stress was applied for 2 d during the uni- and bicellular stages of pollen development. We show that heat stress accelerates pollen development and impairs pollen germination capabilities when applied at the unicellular stage. Heat stress at the bicellular stage impairs sperm cell development and transport into pollen tubes. To understand the course of the latter defects, we used marker lines and analyzed the transcriptomes of isolated sperm cells. Heat stress affected the expression of genes associated with transcription, RNA processing and translation, DNA replication, and the cell cycle. This included the genes encoding centromeric histone 3 (*CENH3*) and *α-tubulin*. Most genes that were misregulated encode proteins involved in the transition from metaphase to anaphase during pollen mitosis II. Heat stress also activated spindle assembly check point and meta- to anaphase transition genes in sperm cells. In summary, misregulation of the identified genes during heat stress at the bicellular stage results in sperm cell development and transport defects ultimately leading to sterility.

## Teaser

Moderate heat stress at the bicellular stage results in defective sperm cells carrying misregulated cytoskeletal, cell cycle, and replication-associated genes in maize.

## Introduction

Global warming is associated with hotter, longer, and more frequent heat waves. When these high temperature episodes occur during reproductive development in crop plants, a significant decrease in yield is commonly observed (Folsom et al. 2014; Chen et al. 2016; Begcy et al. 2019; Nadakuduti et al. 2023). Within reproductive development, male gametophyte formation is one of the most susceptible stages (De Storme and Geelen 2014; Begcy et al. 2019; Chaturvedi et al. 2021). In flowering plants (angiosperms), male gametophyte development undergoes 2 phases, microsporogenesis and microgametogenesis. Four microspores are generated during the first phase from a microspore mother cell undergoing meiosis. During the second phase, 2 rounds of mitotic division give rise to tricellular pollen grains in most angiosperms (Zhou et al. 2017; Hafidh and Honys 2021; Huang et al. 2021). The first division, known as pollen mitosis I (PMI), is highly asymmetric and produces a small generative cell and a large vegetative cell. During pollen mitosis II (PMII), the small generative cell goes through a symmetric division forming 2 sperm cells engulfed by the large vegetative cell that enters into the G0 phase of the cell cycle. Thus, a mature pollen grain in most angiosperms contains 2 small sperm cells attached to the nucleus of the large vegetative cell (Borg et al. 2009; Long et al. 2019; Huang et al. 2021).

In angiosperms including maize (*Zea mays*), sperm cells have lost their motility and are transported as a passive cargo through the pollen tube toward the ovule for double fertilization (Zhou and Dresselhaus 2019). After pollen germination, sperm cells move as a male germ unit (MGU) attached to the nucleus of the vegetative cell before being delivered in the receptive synergid cell and separated (Dumas et al. 1985; Dresselhaus et al. 2016; Sprunck 2020; Sugi et al. 2023). Sperm cell transport and delivery requires a well-organized and dynamic cytoskeleton system (Cheung et al. 2008; Sharma et al. 2021). Fluorescent labeled actin-binding protein and other pollen specific markers have been used to demonstrate that pollen tube growth is especially dependent on the dynamic organization and regulation of actin and its microfilaments (Cheung et al. 2008; Chang et al. 2013; Takeuchi and Higashiyama 2016; Zhang et al. 2023). In tobacco (*Nicotiana tabacum*) and Arabidopsis (*Arabidopsis thaliana*) pollen tubes, it has been shown that heat stress alters cytoskeletal dynamics, isoform content, and spindle orientation (De Storme and Geelen 2013; Parrotta et al. 2016).

The symmetric division occurring during PMII is fundamental for the generation of the 2 sperm cells that will arise from the generative cell either within the pollen grain in most angiosperms or after germination inside the pollen tube like in tobacco and *Amborella trichopoda* (Durbarry et al. 2005; Flores-Tornero et al. 2020; Huang et al. 2021). In *Arabidopsis*, it was shown that sperm cells are not necessary and do not control pollen tube growth and guidance (Zhang et al. 2017).

To ensure accurate chromosome segregation during germ cell division and to produce 2 genetically identical cells during PM II, while further cell division of the vegetative cell is prevented, highly accurate cell cycle checkpoint mechanisms must be executed along these processes. During mitosis, the spindle assembly checkpoint (SAC) is one of these mechanisms that safeguards the transition from metaphase to anaphase (Lara-Gonzalez et al. 2021; Farrell et al. 2023). SAC monitors proper attachment of spindle microtubules to the surface of the kinetochores. In *Arabidopsis*, SAC architecture is different from the one characterized in yeast and animals (Caillaud et al. 2009; Komaki and Schnittger 2016, 2017). Moreover, plant cells are able to reset the cell cycle with duplicated chromosomes avoiding nuclear division (Kozgunova et al. 2019). In maize, some of the major components of the SAC have been identified and showed conserved function (Yu et al. 1999; Du and Dawe 2007; Li and Dawe 2009; Su et al. 2021), but it is unclear whether SAC plays also a role in PM II.

Reduction in fertilization success and yield due to defects in male gametophyte performance has been largely attributed to the lack of pollen viability or inability to germinate under high temperatures (Firon et al. 2006; Endo et al. 2009; De Storme and Geelen 2014; Wang et al. 2017; Begcy et al. 2018, 2019). Low starch content and decreased enzymatic activity, energy, and lipid formation are major cellular components impacted by temperature (Thalmann and Santelia 2017; Yang et al. 2018; Begcy et al. 2019; Kumar et al. 2023). In a previous study, we showed that transient heat stress over 2 d at the tetrad stage has a strong impact on further pollen performance (Begcy et al. 2019). It remained unclear whether successive stages, uni- and bicellular pollen, as well as sperm cell formation and delivery are also affected if heat stress is applied at later stages.

Here, we demonstrate that transient heat stress during the uni- and bicellular stages accelerates pollen development in maize. Moreover, sperm cell formation and sperm transport in the pollen tube is affected ultimately leading to sterility. With a focus on heat stress application during the bicellular stage, we aimed to understand the underlying molecular mechanisms that lead to sterility after comparing the transcriptomes and proteomes of stressed and unstressed pollen grains.

## Results

### Seed set is strongly reduced if heat stress is applied during the uni- or bicellular stage of pollen development

**Figure 1.**
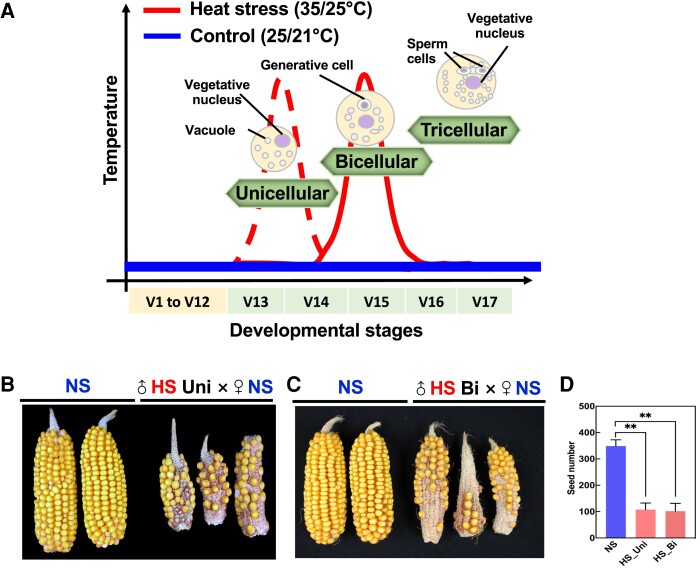
Heat stress during the unicellular and bicellular stages of pollen development reduces seed set in maize. **A)** Experimental setup. Maize plants were grown in control conditions (25 °C/21 °C light/dark period) until they reached either the unicellular or bicellular stage of pollen development. Stages were identified according to Begcy and Dresselhaus (2017). At either stage V13 (unicellular; red dotted line) or V15 (bicellular; red line), plants were exposed to moderate heat stress (35 °C/25 °C light/dark period) for 48 h and afterward transferred back to control conditions until maturity. Blue line indicates (NS) conditions. NS cobs were pollinated with either NS pollen (NS × NS), (**B**) HS pollen at the unicellular stage (♂HS Uni × ♀NS), or (**C**) HS pollen at the bicellular stage (♂HS Bi × ♀NS). Images were digitally extracted for comparison. **D)** Seed set was strongly reduced after pollinating with HS pollen, regardless of the pollen developmental stage in which heat stress was applied. *n* = 15 for panels **B)** and **C)**. Data are presented as the mean ± Sd. Two asterisks indicate significant difference at *P* < 0.001; 1-tailed *t* test comparing HS (red) with NS (blue) samples.

Historically, pollen development studies under heat stress are performed across several developmental stages simultaneously and occasionally through the entire pollen formation process (Firon et al. 2006; Wang et al. 2017; Begcy et al. 2019; Wada et al. 2020). By dissecting the impact of heat stress on a single pollen stage, we previously showed that heat stress during the tetrad stage impacts starch, lipid, and energy metabolism (Begcy et al. 2019). To elucidate whether heat stress triggers similar responses during other stages of pollen development, we imposed heat stress (35 °C/25 °C light/dark period) for 48 h on maize plants specifically either at the unicellular or bicellular stage. Since the transition between maize pollen developmental stages lasts several days (Begcy and Dresselhaus 2017), we applied 48 h heat stress to ensure that we were only targeting a single developmental stage. A parallel set of maize plants was maintained under optimal growth conditions (25 °C/21 °C light/dark period) in an equivalent chamber and was used as a control for all experiments (see Fig. 1A for experimental setup). Since fertilization ability is the most important indicator to evaluate pollen quality, we first pollinated nonstressed (NS) cobs with NS pollen as a control and as expected obtained full seed set (Fig. 1, B and C). In contrast, cobs pollinated with heat-stressed (HS) pollen at either the unicellular (Fig. 1B) or bicellular stage (Fig. 1C) resulted in severe reduction of seed set (Fig. 1, B and C) of ∼65% for both developmental stages (Fig. 1D). Taken together, these results show that heat stress applied during either the unicellular or bicellular stage of pollen development leads to sterility and strongly reduced yield.

### Heat stress at the uni- and bicellular stages accelerates pollen development

**Figure 2.**
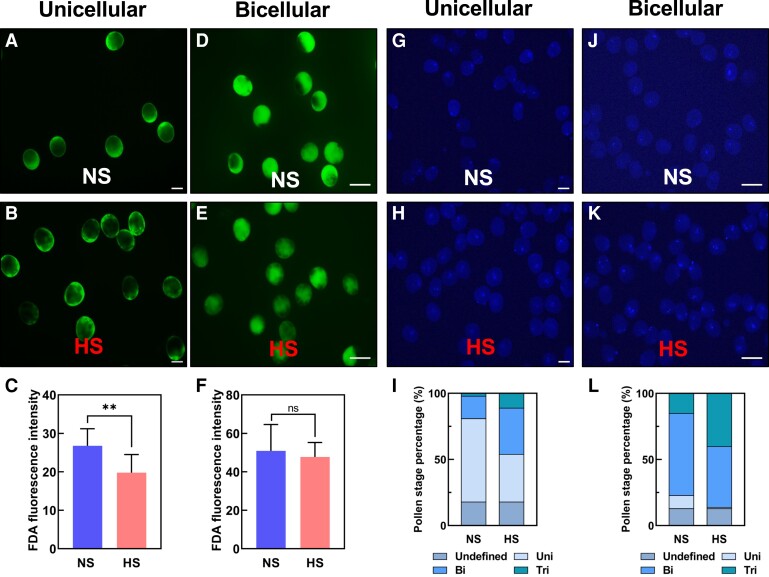
Heat stress at the unicellular and bicellular stages accelerates pollen development. **A)** Pollen stained with FDA after NS and (**B**) HS treatment at the unicellular stage. **C)** Quantification of enzymatic activity of pollen shown in **A)** and **B)**. **D)** Pollen stained with FDA after NS and (**E**) HS treatment at the bicellular stage. **F)** Quantification of enzymatic activity of pollen shown in **D)** and **E)**. **G)** DAPI staining of NS and (**H**) HS pollen at the unicellular stage. **I)** Quantification of pollen shown in **G)** and **H)**. Steel blue, undefined stage; artic blue, unicellular stage; azure blue, bicellular stage; and teal blue, tricellular stage of maize pollen development. **J)** DAPI staining of NS and (**K**) HS pollen at the bicellular stage. **L)** Quantification of pollen shown in **J)** and **K)**. For description, see **I)**. Data are presented as the mean ± Sd. *n* = 400 to 500. Scale bars = 50 *μ*m. Two asterisks indicate significant difference at *P* < 0.001; 1-tailed *t* test comparing HS (red) with NS (blue) samples.

To further explore the possible causes of seed set reduction, we initially analyzed the effect of heat stress on pollen morphology and viability immediately after heat stress application. Similar to what we observed during heat stress application at the tetrad stage, we found decreased pollen viability when HS was applied at the unicellular (Supplementary Fig. S1) but not at the bicellular stage (Supplementary Fig. S2). However, in contrast to heat stress during the tetrad stage, we did not see any morphological alteration after heat stress application at either stage (Supplementary Figs. S1 and S2). Fluorescein diacetate (FDA) was used as a proxy to measure pollen enzymatic activity of pollen stressed at the unicellular stage. In contrast to NS pollen (Fig. 2A), HS pollen showed significantly lower enzymatic activity (Fig. 2, B and C). When we applied heat stress at the bicellular stage of pollen development, we could not see any significant differences between NS and HS pollen after FDA staining (Fig. 2, D to F). To further explore the impact of heat stress on the timing of pollen development, we stained NS (Fig. 2G) and HS pollen (Fig. 2H) at the unicellular stage with DAPI. More than half of the NS pollen (63%) were at the unicellular stage, whereas only 30% of HS pollen were at the unicellular stage (Fig. 2I). A large portion of them progressed to the bicellular (35%) and tricellular (11%) stages (Fig. 2, H and I), respectively.

After DAPI staining (Fig. 2, J and K) at the bicellular stage, we observed that pollen developed in NS conditions were mostly (65%) at the bicellular stage. Pollen at the unicellular (10%) and tricellular (15%) stages were also observed (Fig. 2L). In contrast, HS pollen showed reduced pollen numbers at unicellular (1%) and bicellular stages (46%). Many pollen were already at the tricellular (39%) stage (Fig. 2L). These results indicate that heat stress accelerates the timing of pollen development and has only a minor effect on pollen vitality and only when applied at the unicellular stage.

### Heat stress at the unicellular stage impairs pollen germination capabilities

**Figure 3.**
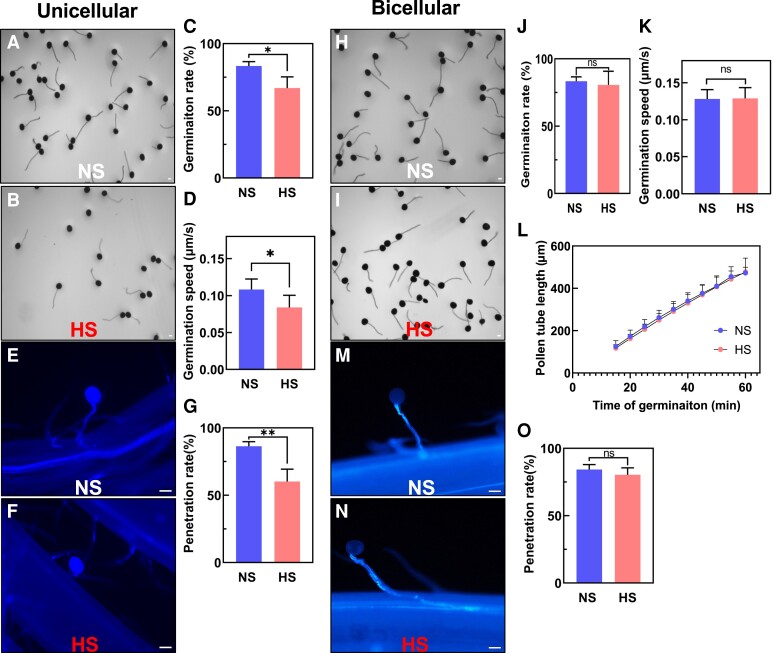
Heat stress applied at the unicellular but not at the bicellular stage impairs pollen germination capabilities. **A)** In vitro germination assays of pollen isolated from NS and (**B**) HS plants at the unicellular stage. **C)** In vitro germination rate of pollen shown in **A)** and **B)**. **D)** Germination speed of NS and HS pollen at the unicellular stage. **E)** Aniline blue staining of NS and (**F**) HS pollen at the unicellular stage germinating on papilla hair cells. **G)** In vivo penetration rate of pollen shown in **E)** and **F)**. **H)** In vitro germination assays of pollen from NS and (**I**) HS plants at the bicellular stage. **J)** In vitro germination rate of pollen harvested from NS and HS plants at the bicellular stage shows no germination rate differences. **K)** Germination speed and (**L**) pollen tube length of NS and HS pollen at the bicellular stage. **M)** Aniline blue staining of NS and (**N**) HS pollen at the bicellular stage germinating on papilla hair cells shows normal pollen tube penetration. **O)** In vivo penetration rate of pollen shown in **M)** and **N)**. Statistically significant differences in germination rate, pollen tube length, and germination speed could not be detected at the bicellular stage between HS and NS conditions. Scale bars = 100 *μ*m. Data are presented as the mean ± Sd. *n* = 400 to 500. One asterisk indicates significant difference at *P* < 0.01; 2 asterisks indicate significant difference at *P* < 0.00; ns indicates no statistical differences. One-tailed *t* test comparing HS with NS samples.

To further explore the impact of heat stress on pollen development during both stages, we transferred HS plants to NS conditions and allowed plants to recover and continue pollen development until maturation. Using mature pollen, we studied in vitro and in vivo germination and pollen tube growth capabilities of both NS and HS pollen at the unicellular (Fig. 3, A to G) and bicellular stages (Fig. 3, H to O). Quantification of in vitro germination rate (Fig. 3C) and speed (Fig. 3D) after heat stress application at the unicellular stage showed a reduction in germination rate of ∼20% (Fig. 3C) as well as a significant reduction in pollen tube growth speed (Fig. 3D). To further confirm these findings, we conducted in vivo germination experiments to investigate the pollen penetration ability on maize silks (Fig. 3, E and F). While NS pollen germinated on silks and penetrated the transmitting track (Fig. 3E), HS pollen at the unicellular stage attached to the silk but were unable to grow a pollen tube into the silk (Fig. 3F). Quantification of the pollen penetration rate showed a 32% reduction when pollen was heat-stressed at the unicellular stage (Fig. 3G). We did not observe such differences in pollen germination and growth capabilities when heat stress was applied at the bicellular stage. Pollen germination rate in vitro (Fig. 3, H to L) and in vivo (Fig. 3, M and N), silk penetration (Fig. 3O), and growth (Fig. 3, K and L) was similar to the NS control (Fig. 3, H to O). In conclusion, the observed sterility (Fig. 1B) when heat stress was applied at the unicellular stage could be explained by pollen germination, growth, and penetration defects. However, sterility caused after HS exposure during the bicellular stage remained unclear.

### Heat stress at the bicellular stage impairs sperm cell development and transport

**Figure 4.**
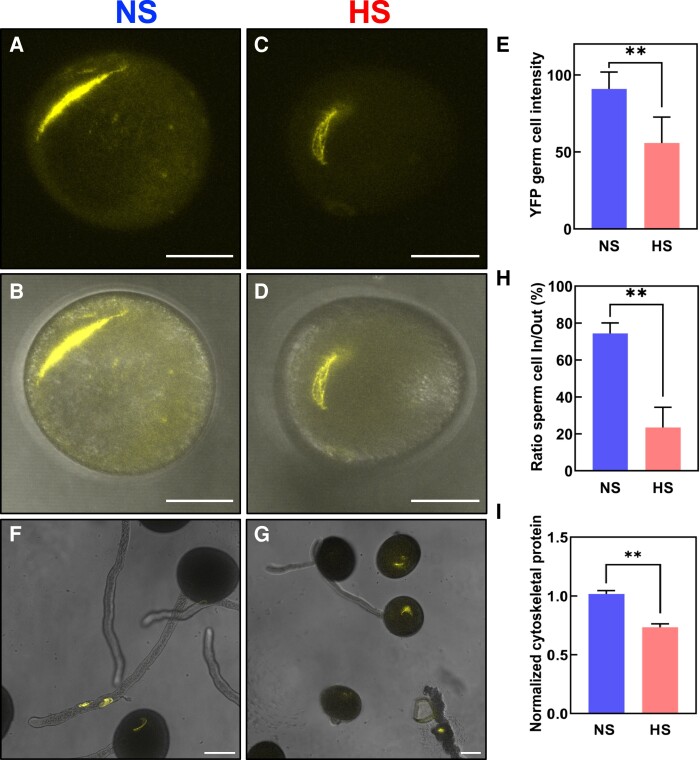
Heat stress at the bicellular stage impairs sperm cell development and transport into the pollen tube. Maize sperm cell maker lines containing α-tubulin fused with YFP were grown in control conditions (25 °C/21 °C light/dark period) until they reached the bicellular stage of pollen development. Then, plants were exposed to heat stress (35 °C/25 °C light/dark period) for 48 h. A parallel set of plants was maintained under control conditions. **A)** Confocal and (**B**) merged images of NS pollen at the bicellular stage. **C)** Confocal and (**D**) merged images of HS pollen at the bicellular stage. **E)** Quantification of YFP signal intensity of pollen as indicated. **F)** Confocal images of NS pollen grains showing sperm cells traveling into the pollen tube. **G)** Sperm cells were kept inside the pollen grain after heat stress was applied at the bicellular stage of pollen development. **H)** Ratio of sperm cells remaining in pollen grain to those traveling inside the pollen tube. **I)** Reduction in the number of detectable cytoskeletal-related proteins in NS and HS pollen at the bicellular stage. See Supplementary Table S1 for details. Asterisks indicate a significant difference at *P* < 0.001; 1-tailed *t* test comparing NS and HS samples. Data are presented as the mean ± Sd. Scale bars = 50 *μ*m. *n* = 400 to 500.

To further elucidate the causes of the strong seed set reduction phenotype without noticeable changes in morphological, cellular, and biochemical properties of HS pollen at the bicellular stage, we next explored the developmental processes occurring at the transition between the bicellular to the tricellular stage of maize pollen development. During the transition to the tricellular stage, the generative cell undergoes mitosis giving rise to 2 sperm cells which during fertilization will fuse with the central cell and the egg cell to form the endosperm and the embryo, respectively (Zhao et al. 2017). Therefore, since the formation of the 2 sperm cells is the main and critical developmental process occurring at the bicellular stage, we hypothesized that heat stress might impact sperm cell development. To test this hypothesis, we used a maize germ and sperm cell marker line that expresses an α-tubulin gene fused with the yellow florescence protein (YFP) and thus labels the spindle apparatus during germ cell division (Kliwer and Dresselhaus 2010). Under NS conditions, the α-tubulin-YFP signal was observed during the mitotic division of the generative cell and marks the 2 spindle-formed sperm cells after division (Fig. 4, A and B). In contrast, the α-tubulin-YFP signal in HS pollen was significantly reduced and the spindle apparatus and sperm cell shape appeared less symmetric (Fig. 4, C and D). Quantification of α-tubulin-YFP signal cells confirmed this observation, and there was a 30% reduction in HS sperm cells compared with NS sperm cells (Fig. 4E). These results demonstrated that even though the morphology of HS pollen did not change, sperm cell formation was significantly impaired.

We next investigated whether this also impacts sperm cell transport inside pollen tubes. We monitored the sperm cell journey using the α-tubulin-YFP marker line and found that within 1 h after pollen germination and growth in vitro, about 80% NS sperm cells were visible inside pollen tubes (Fig. 4, F and H). In contrast, only about 20% sperm cells were visible inside pollen tubes formed by HS pollen (Fig. 4, G and H).

To determine if other cytoskeletal proteins, as well as α-tubulin, were reduced after heat stress, we performed liquid chromatography with tandem mass spectrometry (LC–MS/MS) from mature pollen (Supplementary Table S1). Only proteins that were detected in all 3 biological replicates (“max count 3”) and met the valid threshold of Mascot score > 10 and peptides > 2 in at least one of the conditions (NS and HS) were considered for further analysis. We normalized the number of identified tubulin and actin peptides as well as their associated proteins and noted a significant reduction of tubulin- and actin-related proteins at HS conditions (Fig. 4I). Taken together, our cellular and proteomic data show that heat stress at the bicellular stage impairs sperm cell development and transport due to reduced amounts of cytoskeletal tubulin and actin proteins involved in both sperm cell formation and transport.

### Heat stress at the bicellular stage decreases centromeric histone levels

**Figure 5.**
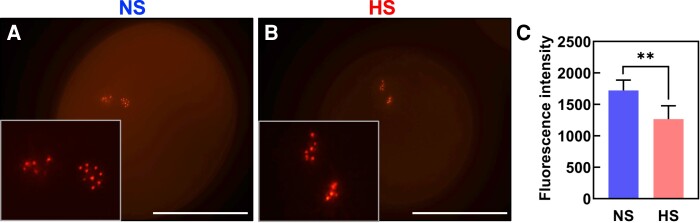
Heat stress during the bicellular stage decreases content of centromeric histones in maize. Marker lines expressing centromeric histones (CENH3) specifically in sperm cells (GEX3p:mRuby3-CENH3) were grown in control conditions (25 °C/21 °C light/dark period) until they reached the bicellular stage of pollen development and then submitted to heat stress (35 °C/25 °C light/dark period) for 48 h. A parallel set of marker line plants was maintained under optimal conditions and used as controls. **A)** Confocal images showing CENH3 in mature pollen grains from NS and (**B**) HS plants. Insets show enlarged sperm cells containing CENH3 signals. Insets were magnified 3×. **C)** Intensity quantification of mRuby3-CENH3 in both conditions. Asterisks indicate a significant difference at *P* < 0.001; 1-tailed *t* test comparing NS and HS samples. Data are presented as the mean ± Sd. Scale bars = 50 *μ*m.

It remained unclear whether reduced tubulin levels and a malformed spindle apparatus lead to chromosome losses and thus sperm cells defects. The centromeric histone H3 variant CENH3 is essential for kinetochore assembly and establishment and thus an ideal marker to label each chromosome showing also equivalent homologous chromosome segregation in eukaryotes (Collins et al. 2005; Keçeli et al. 2020). We generated a sperm cell-specific mRuby3-CENH3 marker by using 1.3 kbp upstream of the sperm cell-specific gamete-expressed 3 (*GEX3*) gene of maize (Chen et al. 2017) as promoter. Ten chromosomes were clearly visible in each sperm cell inside NS pollen grains at maturity due to mRuby3-CENH3 labeling of centromeric chromosome regions (Fig. 5A). However, when the GEX3p:mRuby3-CENH3 marker line was exposed to HS conditions during the bicellular stage of pollen development, fluorescent signals at some chromosomes appeared weaker, although a comparable number of chromosomes could be observed (Fig. 5B). Quantification of mRuby3-CENH3 signal showed a significant reduction in HS pollen compared with NS conditions (Fig. 5C). These results indicate that CENH3 levels are reduced after heat stress and appear more irregular at centromeric regions indicating sperm cell defects.

### Heat stress impacts expression of genes involved in transcription, DNA replication, RNA processing, and translation in sperm cells

**Figure 6.**
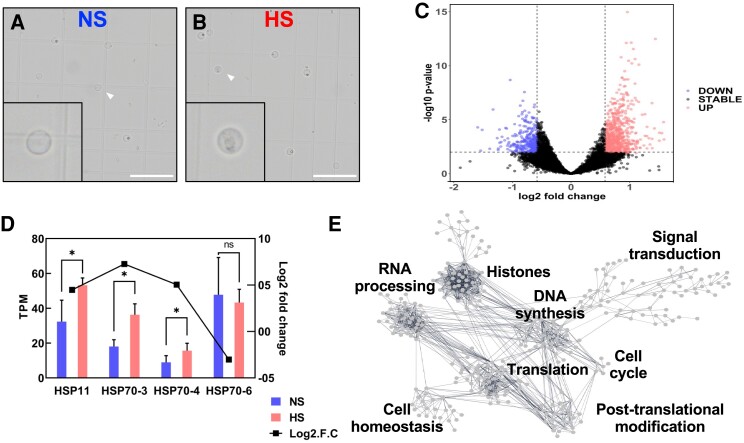
Heat stress at the bicellular stage misregulates replication-associated genes in sperm cells. **A)** Confocal images of NS and (**B**) HS sperm cells at the bicellular stage of pollen development. Insets show enlargement of single sperm cells (arrowheads). Insets were magnified 4×. Scale bar = 50 *μ*m. Approximately 5,000 individual sperm cells in each of the 3 biological replicates per condition were used. **C)** Differential gene expression (base 2 logarithm fold change) in sperm cells harvested at maturity after heat stress was applied at the bicellular stage of pollen development. HS samples relative to control (NS) samples were plotted versus average gene expression levels (i.e. logarithm of mean counts normalized for difference in library sizes). Red color indicates upregulation. Blue color indicates downregulation. Black color indicates no significant transcriptional change. **D)** TPM values and RT-qPCR analysis show that differential expression of HSP genes in sperm cells was still increased in mature pollen. Asterisks indicate significant difference at *P* < 0.01; 1-tailed *t* test comparing HS with NS samples. *n* = 3 biological replicates, each with 3 technical replicates. Data are presented as the mean ± Sd. **E)** Gene network analysis of interactions of differentially expressed genes in sperm cells in response to heat stress at the bicellular stage. A threshold of 0.7 edge confidence was used. A detailed list of the genes included in the gene interaction analysis can be found in Supplementary Table S4.

To elucidate the molecular mechanisms impacted by heat stress during maize sperm cell development, we used an RNA sequencing (RNA-seq) approach to further analyze the transcriptional changes imposed by increased temperatures at the bicellular stage. Maize sperm cells were isolated using a Percoll gradient strategy (Dupuis et al. 1987; Chen et al. 2017). We isolated ∼5,000 individual sperm cells in each of the 3 biological replicates of both NS and HS samples. Heat stress was applied for 48 h at the bicellular stage, and then, plants were maintained under control conditions until they reached pollen maturation (Fig. 6, A and B; Supplementary Table S2). We found 952 genes differentially expressed between NS and HS conditions (Fig. 6C). Among these genes, 267 were downregulated and 684 were upregulated (Supplementary Table S3). Gene ontology analysis showed enrichment of genes associated with chromosome and chromatin organization, DNA conformation, cellular homeostasis, and purine-containing compound metabolic process (Supplementary Fig. S3). Previously, we showed that heat stress at the tetrad stage of maize pollen development induced the expression of several heat shock protein (HSP) genes, which were still detectable in pollen at maturity (Begcy et al. 2019). Members of the *HSP70* family have been shown to be expressed during pollen development (Gagliardi et al. 1995). Thus, we compared expression of HSP genes in sperm cells at both conditions. An overall low or lack of expression of HSP and heat shock factor (HSF) genes has been reported in NS sperm cells of maize (Chen et al. 2017; Warman et al. 2020). After HS application, we found a significant transcriptional increase only in *HSP11* (Zm00001eb011930), *HSP70-3* (Zm00001eb012510), and *HSP70-4* (Zm00001eb136490) (Fig. 6D). *HSP70-6* (Zm00001eb012470) was not induced in sperm cells after heat stress at the bicellular stage and was used as a control (Fig. 6D). These results suggest that short spikes of heat stress likely do not interfere significantly with the gene regulatory network of sperm cells.

To further understand sperm cell development-associated genetic responses to heat stress, we used the identified differentially expressed genes and conducted a cluster correlation analysis to elucidate functional groups of genes with shared expression profiles. We merged our cluster correlation analysis with the web-tool STRING (Szklarczyk et al. 2019), to identify genes previously described as members of any given pathway. First, we removed unrooted genes and identified 331 interacting genes associated with the heat stress response in sperm cells (Supplementary Table S4). To select candidate genes in response to heat stress from sperm cells, we used a stringent edge confidence of at least 0.7. Previously using this approach, we found 5 main clusters after heat stress during the tetrad stage of pollen development (Begcy et al. 2019). Our current analysis yielded 8 main hubs affected by heat stress in sperm cells (Fig. 6E). Notably, these hubs are formed by genes related to histones, DNA synthesis, RNA processing, translation, posttranslational modification, cell cycle, and signal transduction (Fig. 6E). Notably, the misregulation of histone genes confirms the previous observation that levels of centromeric histone CENH3 are reduced after heat stress (Fig. 5).

### Heat stress affects highly expressed genes in sperm cells

To compare the transcriptional status of NS and HS sperm cells, we analyzed the genome wide transcript per million (TPM) values of both conditions (Supplementary Fig. S4). When TPM values from the entire maize genomes were plotted, a high correlation (*R*² = 0.9868) in gene expression was obtained between both conditions (Supplementary Fig. S4A), suggesting that even though heat stress at the bicellular stage impacts the expression of genes in sperm cells, the level of changes appeared minor. However, this analysis also included a larger portion of genes that are not expressed in any of the conditions. Our sperm cell analysis yielded an average of 30% transcriptional expression of the entire maize genome in both conditions. No significant differences were observed when genes >1 and <100 TPM were used (Supplementary Fig. S4B). Therefore, we used a 100 TPM cutoff, so that only highly expressed genes were used in the analysis, and a lower correlation of TPM levels (R² = 0.7477) was found (Supplementary Fig. S4C). We further dissected the transcriptional response to heat stress using 150 TPM (Supplementary Fig. S4D) and 200 TPM (Supplementary Fig. S4E) cutoffs, respectively, and found a significant impact when highly expressed genes were included. Our results suggest that heat stress at the bicellular stage has a large effect mostly on highly expressed genes and therefore critical for sperm cell development.

### Heat stress misregulates cell cycle control genes in sperm cells but does not affect their DNA content

**Figure 7.**
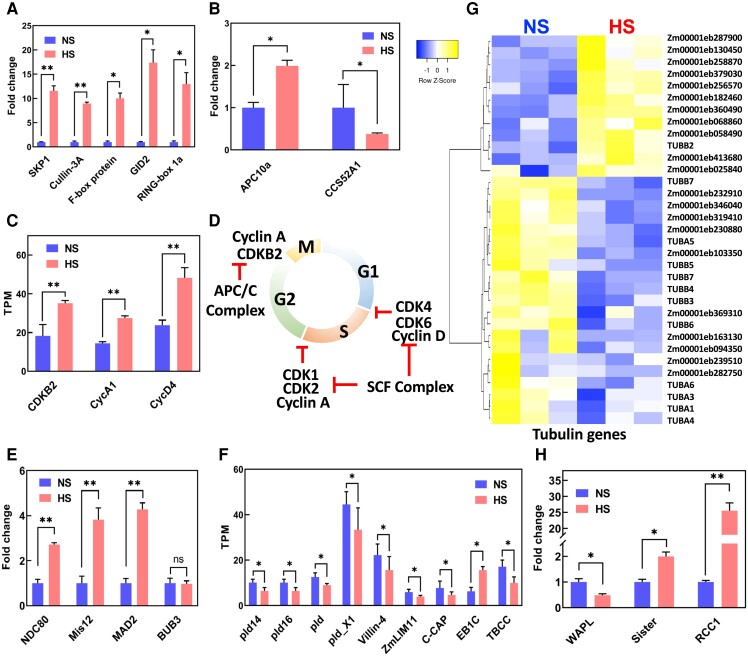
KP1-CUL1-F-box protein (SCF) E3 ubiquitin ligase complex and spindle assembly check point (SAC) genes are upregulated in sperm cells after heat stress was applied during the bicellular stage of pollen development. **A)** Induction of the SCF member genes. **B)** Misregulation of the APC/C complex genes. **C)** Upregulation of cyclins. **D)** Illustration of the cell cycle during PM II and its regulation by cyclins and the SCF complex. **E)** Upregulation of SAC gene members after heat stress. NDC80, component of the kinetochore complex. **F)** Downregulation of microtubule-associated genes. **G)** Misregulation of tubulin-associated genes. **H)** Misregulation of genes associated with mitosis progression during the metaphase-to-anaphase transition. WAPL, wings apart-like protein homolog; RCC1, regulator of chromosome condensation 1; Sister, sister chromatid cohesion 1. One asterisk indicates significant difference at *P* < 0.01; 2 asterisks indicate significant difference at *P* < 0.001; 1-tailed *t* test comparing NS and HS samples. *n* = 3 biological replicates, each with 3 technical replicates. Data are presented as the mean ± Sd.

We next explored in more detail which genes are differentially regulated by heat stress during sperm cell formation. In plants, the SCF E3 ligase complex formed by the S-phase kinase-associated protein1 (*SKP1*; Zm00001eb404320), cullin3A (*CUL3A*; Zm00001eb254590), an F-box protein (Zm00001eb187770), F-box protein gibberellin-insensitive dwarf2 (*GID2*; Zm00001eb245180) and ring-box protein 1A (*RBP1*; Zm00001eb188540) plays a key role integrating developmental end environmental responses (Ban and Estelle 2021). Notably, all genes encoding members of the SCF complex showed upregulation of more than 10 times (Fig. 7A). Most genes encoding members of the anaphase-promoting complex/cyclosome APC/C, another multimeric E3 ligase, were not expressed in sperm cells (Supplementary Table S5). However, anaphase-promoting complex (*APC10a*; Zm00001eb342630) and cell cycle switch protein 52 A1 (*CCS52A1*; Zm00001eb001710) were misregulated after heat stress (Fig. 7B). *SAMBA* (Zm00001eb024740) and *CCS52B* (Zm00001eb139790), which encode other members of the APC/C complex, showed expression in sperm cells, but their transcript levels did not change in response to heat stress (Supplementary Table S5). Since the SCF and APC/C complexes regulate cyclins and cyclin-dependent kinases (CDKs) controlling the progression of cell cycle, we searched for maize CDK genes whose expression pattern changed in response to heat stress. Out of the total number of cyclins and CDKs described in maize, only 3 genes were differentially expressed in maize sperm cells after HS: cyclin A1 (*ZmCycA1*; Zm00001eb227730), cyclin D4 (*ZmCycD4*; Zm00001eb038430) and cyclin-dependent kinase B2 (*ZmCDKB2*; Zm00001eb035350) (Fig. 7C). All 3 genes were induced. Noteworthy, these 3 genes were also the only ones expressed in maize sperm cells under control conditions. In summary, we found that heat stress induced upregulation of genes associated with the SCF and APC/C E3 ligase complexes, which are known to control the degradation of cell cycle regulators (cyclins and CDKs) to allow G1-to-S transition as well as S-to-G2 transition (Kim et al. 2008; Villajuana-Bonequi et al. 2019) and thus promote cell cycle progression (Fig. 7D).

We next measured the DNA content of sperm cells to elucidate whether misexpression of cell cycle regulator genes may affect DNA replication and ploidy level of sperm cells. In *Arabidopsis*, it has been shown that cold and heat stress disrupts genomic ploidy consistency, generating the formation of diploid and polyploid pollen (De Storme et al. 2012; Lei et al. 2020). Therefore, we performed comparative ploidy-level measurements by using flow cytometry on sperm cells isolated from mature pollen previously heat-stressed at the bicellular stage. As a control, we used nuclei isolated from leaf tissue and observed the 2C and 4C peaks that are typical for this type of tissue indicating diploidy (Supplementary Fig. S5A). Only 1 dominant 1C peak was observed in NS sperm cells (Supplementary Fig. S4B). Similarly, sperm cells from HS pollen showed 1 single and sharp 1C peak indicating haploidy and lack of aneuploidy (Supplementary Fig. S5C). These findings demonstrate that sperm cells still contain a normal DNA content, but it does not exclude DNA/chromatin defects that occurred due to improper cell cycle checkpoint controls.

### Heat stress activates spindle assembly check point and meta- to anaphase transition genes in sperm cells

Since our data point toward misregulation of genes associated with PM II, we searched for developmental transitions involved in this process. During the transition from metaphase to anaphase, where chromosomes aligned at the equator of the cell and migrate to the poles, a developmental process ensures that all chromosomes are aligned at the same level. This process is controlled by the SAC signaling complex, which regulates proper partitioning of chromosomes to daughter cells during mitosis. SAC signaling is a mechanism that it is only active when chromosomes are not properly attached to the kinetochores and thus delays progression of the cell cycle until all kinetochores are correctly assembled (Lara-Gonzalez et al. 2021). We found upregulation of genes encoding nuclear division cycle 80 (Ndc80, Zm00001eb040130), minichromosome instability 12 (Mis12, Zm00001eb433940), and mitotic arrest deficient 2 (Mad2, Zm00001eb425240) in sperm cells generated during heat stress treatment (Fig. 7E). These proteins are part of the SAC signaling pathway. Expression of a gene encoding another member of SAC signaling, budding uninhibited by benzimidazoles 3 (BUB3, Zm00001eb151660), did not change in response to heat stress (Fig. 7E). In conclusion, these results indicate that heat stress activates SAC signaling and possibly also the generation and regulation of the spindle apparatus. Therefore, we searched for microtubule-related genes in our sperm cell data sets. We identified 9 microtubule-related genes with TPM vales higher than 5 and classified them into 2 groups: microtube-related signaling and microtubule functions (Fig. 7F). Four of the microtube-related signaling genes belong to the phospholipase D family. Phospholipase D activity has been associated with many physiological processes including organization of the cytoskeleton, endocytosis, and exocytosis but also stress responses during development and immunity (Ali et al. 2022). All 4 phospholipase D genes were downregulated in response to heat stress (Fig. 7F). Similarly, the gene encoding VILLIN3 (Zm00001eb389350), which is required for the generation of actin filament bundles (Van Der Honing et al. 2012), was downregulated after heat stress at the bicellular stage of pollen development. Another microtube-related signaling gene encodes a LIM (lineage-11 [LIN-11], insulin-1 [ISL-1], and mechanotransduction-3 [MEC-3]; Zm00001eb268250) gene. LIMs are cytoskeleton-associated proteins that inhibit actin filament depolymerization and cross-link filaments in bundles (Thomas et al. 2007). Downregulation of this gene was also found after heat stress (Fig. 7F). Another gene in the microtube-related signaling category encodes an adenylyl cyclase-associated protein (C-CAP, Zm00001eb040800). Similar to the other genes, its gene expression downregulated in response to heat stress (Fig. 7F). The only 2 differentially expressed genes encoding proteins involved directly in microtubule function showed opposite transcriptional expression: while the microtubule-associated gene end binding protein 1C (*EB1C*; Zm00001eb044540) was upregulated, tubulin binding cofactor C (*TBCC*, Zm00001eb236410) was downregulated in HS sperm cells (Fig. 7F). We further explored a full set of structural tubulin genes differentially expressed after heat stress and found that more than 63% of them were downregulated (Fig. 7G; Supplementary Table S6).

Another set of proteins important for the transition from metaphase to anaphase are wings apart-like protein homolog (WAPL, Zm00001eb220920), regulator of chromosome condensation 1 (RCC1; Zm00001eb049950), and sister chromatid cohesion 1 (Sister; Zm00001eb412050) (Fig. 7H). WALP induces cohesin dissociation from DNA allowing the progression of the mitotic cell cycle, which is modulated by Sister (Crawley et al. 2016). We found that *WALP* was downregulated after heat stress, while *Sister* is upregulated (Fig. 7H). *RCC1*, another important gene whose product associates with chromatin dynamically controlling metaphase-to-anaphase transition, was highly upregulated after heat stress (Fig. 7H). Collectively, our results show that heat stress impacts sperm cell development by targeting spindle assembly checkpoint and metaphase-to-anaphase transition genes whose misregulation might cause defective sperm cells.

## Discussion

Heat stress directly affects plant reproduction processes by decreasing the viability of reproductive cells ultimately leading to fertilization failures and sterility and thus declining plant productivity (Chen et al. 2016; Begcy et al. 2019; Chaturvedi et al. 2021; Kumar et al. 2023; Nadakuduti et al. 2023). Here, we show that HS applied exclusively either at the unicellular or bicellular stage of pollen development results in a strong reduction in seed set and yield. Reduction in seed set upon exposure to high temperatures during the plant reproduction phase has been observed in many field crops (Prasad et al. 2017; Sun et al. 2018; Begcy et al. 2019; Wang et al. 2020), but the underlying molecular mechanisms affected are largely unclear. Heat stress at the unicellular stage resulted in lower enzymatic activity, reduction of germination rate, as well as a decrease in pollen tube penetration rate and growth speed. These phenotypes resemble the ones previously reported on the effect of heat stress applied at the tetrad stage of pollen development in maize (Begcy et al. 2019). Therefore, it is likely that the pollen phenotypes after heat stress at the unicellular stage are also associated with misregulation of starch, lipid, and energy biosynthesis-related genes. Defects in pollen viability and tube growth are common alterations after heat stress leading to a decrease in productivity (Endo et al. 2009; Begcy et al. 2019). However, our results showed that imposing heat stress during the bicellular stage of pollen development in maize did not result in noticeable changes in morphology, vitality, and germination capabilities (Figs. 3 and 4).

Formation of the 2 sperm cells is the central developmental process occurring at the bicellular stage. Disruption of spermatogenesis in mammals due to heat stress has been shown to negatively impact male reproduction altering sperm morphology and leading to DNA fragmentation (Hansen 2009; Garcia-Oliveros et al. 2020; Capela et al. 2022). In plants, little is known on the impact of high temperatures for sperm cell formation. In *Arabidopsis*, spermless pollen tubes were obtained after knocking out 2 basic helix–loop–helix (bHLH) transcription factor genes, *defective region of pollen 1* (*drop1*) and *drop2* (Zhang et al. 2017), indicating that proper pollen tubes can be formed lacking sperm cells but probably also containing defective and nonfunctional sperm cells. Pollen tubes in maize are also formed after heat stress application at the bicellular stage, but sperm cells were not transported. We have shown that tubulin and other cytoskeletal protein genes are misregulated and protein levels reduced after heat stress. Notably, a decline in the number of functional proteins, particularly those involved in cytoskeletal organization, was previously also shown in mature HS tomato (*Solanum lycopersicum*) pollen (Keller et al. 2018). While transport of sperm cells and vesicles in pollen tubes is largely actin-dependent, microtubules and their kinesin motors play among others an important role of linking the sperm cells with the vegetative nucleus forming the MGU (McCue et al. 2011; Cai 2022). Their misexpression can well explain the observed transport defect.

Among the molecular mechanisms that ensure proper sperm cell development is the SAC complex. The SAC is a surveillance mechanism that ensures error-free chromosome segregation by blocking the metaphase-to-anaphase transition during mitosis and meiosis. It is only active when chromosomes are not correctly attached to spindle microtubules emanating from opposing poles (Komaki and Schnittger 2016, 2017; Lara-Gonzalez et al. 2021). We found that heat stress activates SAC signaling in sperm cells. Since the SAC is likely also necessary to ensure genome stability during sperm cell formation (PM II), it is not unlikely that its misregulation may lead to chromosome defects. Our flow cytometry data indicated that these cannot be dramatic as the ploidy level of sperm cells remained unchanged and aneuploidy could not be detected using this method. This could also be because of a phenomenon called mitotic slippage or SAC adaptation. During mitotic slippage, the control of the SAC to delay mitosis disappears if the time taken to form proper chromosomal attachments to spindle microtubules is too prolonged. Thus, the mitotic process continues, and the cells will eventually divide. However, abnormal daughter cells are produced due to genomic and genetic instability (Burgess et al. 2014; Sinha et al. 2019). Since mature pollen grains after heat stress application at the bicellular stage generate functional tubes and contain 2 sperm cells, but seed formation is highly reduced, it is not unlikely that defective sperm cells are the cause due to genome/chromosome defects that occurred during HS at PM II. Genomic sequencing of single sperm cells could now elucidate whether the sperm cells contain defective DNA and/or chromosomes.

Other important components for functional sperm cell development are the cytoskeleton and microtubule organization. In *Arabidopsis*, high temperature conditions were shown to interfere with the configuration of α-tubulin, affecting the construction of the spindle and phragmoplast during male meiosis I and II (Lei et al. 2020). Similarly, in tobacco (*N. tabacum*) cells, heat stress affects the microtubules of the mitotic spindle and phragmoplast, resulting in split spindles, altered microtubule asters, and elongation of the phragmoplast (Smertenko et al. 1997). Notably, when microfilaments essential for sperm nuclear migration in the egg cell cytoplasm were disrupted in *Arabidopsis*, the sperm cell nucleus failed to fuse with either the central and egg cell nucleus, respectively (Kawashima et al. 2014). In *cenh3 Arabidopsis* mutants, high temperature induced pollen reprogramming development to an embryogenic one by reorganization of microtubules (Ahmadli et al. 2023). This suggests that as the temperature increases, the stability of microtubules and chromosomes decreases, as evidenced also in this study by the reduced signal intensity of the centromeric histone CENH3 marker line under heat stress. Of course, also other genes like those required for proper transcription and translation were misregulated after heat stress explaining reduced protein levels and supporting the hypothesis that nonfunctional sperm cells are formed. This has to be investigated in future experimentation, which is still a very challenging task in species containing thick female tissues like maize.

In summary, we show that heat stress applied at the bicellular stage of pollen development leads to misregulation especially of cell cycle regulatory genes and likely impairs also progression during the metaphase-to-anaphase transition by the hyperactivation of SAC, which altogether probably causes defective sperm cells and their MGUs that cannot be properly transported in the pollen tube and do not arrive at the female gametophyte to execute double fertilization. Determining whether and how misexpression of the described genes is avoided in HS tolerant plants will be an exciting task for the future.

## Materials and methods

### Plant material, reporter lines, and growth conditions

In this study, maize (*Z. mays*) inbred line B73 and sperm cell-specific α-tubulin-YFP (Kliwer and Dresselhaus 2010) as well as mRuby3-CENH3 marker lines were used for heat stress studies. The centromeric mRuby3-CENH3 (GEX3p:mRuby3-CENH3) line was generated as follows: 1.3 kbp upstream of the open reading frame (ORF) of the sperm cell-specific *ZmGEX3* gene (Chen et al. 2017; gene ID Zm00001eb161540) was cloned together with the ORF of a mRuby3-CENH3 fusion protein gene into the vector pTF101.1 provided by the maize transformation platform at Iowa State University. Transgenic maize plants were generated using hybrid embryos of inbred lines Hi-IIA and Hi-IIB. All maize seeds were germinated in an incubator, transferred after 10 d to a greenhouse into larger pots (10 cm diameter, 10 seedlings per pot) containing a standard substrate and soil mixture (1:1, *v*/*v*). After 3 wks, maize seedlings were planted into 10 L pots grown under controlled conditions of 16 h of light at 26 °C ± 2 °C and 8 h of darkness at 21 °C ± 2 °C and a constant air humidity of 60% to 65%. Supplemented light of ∼20,000 lux was provided to adjust day length duration. An automated temperature/water-based irrigation system was used to supply water according to plant consumption in a time-based preprogrammed schedule. Plants were fertilized twice a week with 2% (*w*/*v*) Hakaphos and monitored throughout their vegetative and reproductive development. Marker line plants were genotyped before transfer into larger pots. All plants were monitored throughout their entire vegetative and reproductive development.

### Heat stress treatment at the unicellular and bicellular stages

Heat stress was applied at the uni- and bicellular stages of maize pollen development. Identification of pollen developmental stages was performed using the Leaf Collar Method as described previously (Begcy and Dresselhaus 2017) containing between 80% and 95% pollen of the respective stages. After reaching either of the stages, maize plants were transferred to walk-in growth chambers. For heat stress, growth chamber day/night temperature conditions were set at 35° for 16 h light and 25 °C darkness with 60% air humidity at 25,000 lux for 48 h. Correspondingly, NS plants were maintained at a 25 °C/21 °C day/night temperature regime and 60% humidity at 25,000 lux in a control chamber. After 48 h exposure to heat stress, all plants were maintained under control conditions as described above until pollen maturation. Pollen was either collected directly afterwards for morphological and physiological analyses or at pollen maturity for germination and seed set assays. Samples for biochemical and RNA-seq analysis were also collected from pollen at maturity.

### Pollen germination assays

Maize plants used for pollen germination experiments were placed close together to maintain similar conditions and to ensure high quality of pollen harvested. For in vitro pollen germination, solid medium was prepared by mixing 2× pollen germination medium (PGM: 20% sucrose [*w*/*v*]; 0.005% H_3_BO_3_ [*w*/*v*]; 20 mM CaCl_2_; 0.1 mM KH_2_PO_4_; 12% PEG4000 [*w*/*v*], pH = 5) and an equal volume of autoclaved 1.2% NuSieve GTGTM agarose (*w*/*v*) (Lonza) to a final agarose concentration of 0.6% (*w*/*v*). A total of 3 mL of PGM medium mixture was pipetted into a 35 mm petri dish and gently agitated horizontally to obtain a thin and evenly distributed medium layer after 10 min solidification at room temperature (RT). Freshly collected pollen from NS and HS plants was obtained by softly shaking new released florets into petri dishes containing solid PGM. Pollen was germinated at RT (22 to 23 °C) in a wet and dark chamber. The pollen germination status was monitored after 40 to 45 min on PGM and visualized using a Nikon Eclipse 1500 microscope with a 4× objective (Plan Fluor DL 4×/0.13, PHL) equipped with a Zeiss AxioCam MRM monochromatic camera.

In vivo pollen germination was carried out as previously described (Begcy et al. 2019) with some modifications. Before silking, ears from NS maize plants were covered using small paper bags to prevent pollen contamination. Fresh pollen grains from NS and HS plants were harvested using a paper bag and pollinated on newly emerged silks. After 1 h of in vivo pollen germination, 5 cm from the top portion of pollinated silks were cut off and fixed in 9:1 *v*/*v* ethanol:acetic acid at 4 °C overnight. Fixed samples were rehydrated by a water series. Then, silks were treated with 8 M sodium hydroxide for 2 to 4 h to clear and soften the tissue. Softened silks were washed 2 to 4 times using water. Staining was carried out using aniline blue staining solution (0.1% aniline blue [*v*/*v*]; 0.1 M K_2_HPO_4_·3H_2_O, pH = 11) overnight at 4 °C. Samples were washed and mounted using fresh staining solution on a slide with a cover slip and analyzed under fluorescence microscope ZEISS Axio Imager 2 with a 20× objective (Plan-Apochromat 20×/0.8 M27) at 350∼400 nm (UV) excitation.

### Maize sperm cell isolation

A discontinuous percoll density gradient centrifugation method was used as previously described (Dupuis et al. 1987; Chen et al. 2017) with some modifications. Fresh pollen grains from NS and HS B73 maize plants were harvested and placed into glass petri dishes containing a moist filter paper in the internal part of the lid. Pollen was allowed to prehydrate at RT for at least 2 h. Then, pollen was immersed in 550 mOsmol/kg H_2_O mannitol solution (100 mg pollen/mL solution) and incubated on a platform shaker with slow agitation (80 rpm) for 1 to 2 h. Pollen lysates were filtered in a 50 mL Falcon conical tube equipped with a pluriStrainer 30 *µ*M polyester mesh (PluriSelect) and a connector ring (PluriSelect) allowing force filtering of samples using manual pressure with a syringe. Subsequently, a cleared lysate obtained from the filtering step was layered on top of a discontinuous 3-phase percoll gradient consisting of 30%/20%/15% (*v*/*v*) percoll in 550 mOsmol/kg H_2_O mannitol solution. Centrifugation was performed at 12,000 × *g* for 1 h at 4 °C. After density gradient centrifugation, the 20%/30% interphase, where sperm cells accumulated as a faint yellowish line, was collected using a Pasteur pipette and transferred to a 10× volumes 550 mOsmol/kg H_2_O mannitol solution, followed by another centrifugation at 2,500 × g to wash out pollen organelles and other cytoplasmic contaminants. Pelleted sperm cells were resuspended in 20 *μ*L of 550 mOsmol/kg H_2_O mannitol solution, and the number of isolated sperm cells was assessed using a cell counting chamber (Marienfeld). Isolated sperm cells were used immediately or shock-frozen in liquid nitrogen and stored at −80 °C.

### RNA isolation and RT-qPCR

To ensure high RNA quality, all tubes and tips used during pollen RNA extractions were RNase-free and metal beads were autoclaved. Working bench and pipettes were cleaned with RNase Zap RNase Decontamination Solution (Thermo Fisher Scientific). Total RNA was isolated from NS and HS sperm cells or pollen grains using the Invitrogen TRIzol Plus RNA Purification Kit (Thermo Fisher Scientific). Approximately 0.2 g pollen were each collected in a 2 mL microcentrifuge tube with 1 metal ball inside. Samples were frozen in liquid nitrogen and ground to powder using a TissueLyser II (Qiagen). One mL of TRIzol reagent was added to each sample and vortexed adequately. After 5 min incubation at RT, 200 mL of chloroform was added to each sample. After inverting several times and incubation for 2 to 3 min, samples were centrifuged for 15 min at 12,000 × *g* at 4 °C. Around 600 *µ*L upper colorless layer supernatant containing RNA was transferred to a new tube and mixed with equal volume of 70% ethanol (*v*/*v*) by inversion. The mixture was transferred into spin cartridges provided by the kit and centrifuged at 12,000 × *g* for 15 s. To remove DNA contamination, RNase-free DNase solution (Qiagen) was added to each column and centrifuged. RNA on the membrane was washed by sequential washing buffers supplied with the kit. Total RNA was eluted out from membranes by adding 50 *µ*L prewarmed 60 °C RNase-free water for obtaining high RNA yield. For quantification and quality control, isolated RNA was photometrically analyzed using the NanoDrop ND 100 system (Thermo Fisher Scientific) and stored at −80 °C. Complementary DNA (cDNA) synthesis was performed using 1 *μ*g of total RNA using Oligo (dT) 18 primer and reverse transcriptase RevertAid (Thermo Fisher Scientific) according to instruction as previously described (Preciado et al. 2022). For normalization, an ubiquitin gene (gene ID Zm00001eb066940) was used as a control. PCRs were performed using the Master Cycler realPlex2 (Eppendorf) in a 96-well reaction plate according to the manufacturer’s recommendations. Primers are listed in Supplementary Table S7. Cycling parameters consisted of 5 min at 95 °C followed by 40 cycles of 95 °C for 15 s, 60 °C for 30 s, and 70 °C for 30 s. Reactions were performed in triplicate for each RNA sample using at least 3 biological replicates. Specificity of amplifications was verified by melting curve analyses. Results from the Master Cycler realPlex2 detection system were further analyzed using Microsoft Excel (Kim et al. 2021). Relative amounts of mRNA were calculated from threshold points (Ct values) located in the log-linear range of RT-qPCR amplification plots using the 2- ^ΔΔCT^ method (Livak and Schmittgen 2001).

### DAPI microscopy

Isolated pollen grains at different stages using the Leaf Collar Method (Begcy and Dresselhaus 2017) were placed on glass slides containing 1 mg/mL DAPI solution in 1× phosphate-buffered saline (PBS; 0.8% [*w*/*v*] NaCl, 0.002% [*w*/*v*] KCl, 0.014% [*w*/*v*] Na_2_HPO_4_, and 0.0024% [*w*/*v*] KH_2_PO_4_) and observed using a Zeiss Axio Imager Z1 microscope equipped for structured illumination (Apotome2) and with a Zeiss AxioCam MRM monochromatic camera. DAPI fluorescence was observed using a filter set with a wavelength of 359 nm.

### Pollen vitality assays

Freshly harvested mature pollen from NS and HS plants were subjected to cytological analysis to assess their metabolic activity. Pollen grains were stained with FDA as previously described (Begcy et al. 2019) and with Alexander staining then visualized using a Zeiss Axio Imager Z1 microscope (Apotome) equipped with a fluorescein isothiocyanate (FITC) filter, which allows the detection of fluorescein. Excitation and emission filters were set at 450 and 520 nm wavelength, respectively (Zeiss). Pollen grains that exhibited fluorescence in the FITC channel were considered metabolically active.

### Microscopy of α-tubulin-YFP as well as mRuby3-CENH3 marker lines

For imaging of the α-tubulin-YFP marker line at the bicellular stage of pollen development under NS and HS conditions, anthers of both conditions were harvested right after heat stress treatment and directly embedded in tubes containing 1× PBS (0.8% [*w*/*v*] NaCl, 0.002% [*w*/*v*] KCl, 0.014% [*w*/*v*] Na_2_HPO_4_, and 0.0024% [*w*/*v*] KH_2_PO_4_). Released pollen were observed using a confocal microscope (Leica TCS SP8) with a Plan-Apochromat 40×/1.3 Oil DIC objective and visualized using the Leica Application Suite X software. Fluorescence signals were detected with excitation at 514 nm and emission between 525 and 600 nm. The gain value was set at 500 and pinhole was set always at 1 AU (65.3 *μ*m). Images were captured by Z-stacks with interplane interval at 0.4 *μ*m. For imaging of the mature pollen of the mRuby3-CENH3 marker line, fresh mature pollen under NS or HS conditions at the bicellular stage of pollen development was harvested and observed using a Zeiss Axio Imager Z1 microscope equipped for structured illumination (Apotome2) and with a Zeiss AxioCam MRM monochromatic camera by using ZEN3.1 Blue software (Zeiss). mRuby3 fluorescence was observed using a filter set with a wavelength of 558 nm. Images were captured by z-stacks with interplane interval at 0.5 *μ*m. Quantification of intensity was measured using the Fiji ImageJ platform (Schindelin et al. 2012).

### Library preparation and Illumina RNA-seq

Library preparation and RNA-seq were conducted following the Illumina TruSeq Stranded mRNA Sample Preparation Guide, the Illumina HiSeq 1000 System User Guide (Illumina), and the KAPA Library Quantification Kit-Illumina/ABI Prism User Guide (Roche). In brief, 250 ng of total RNA of NS and HS sperm cells were used for purifying polyA^+^ containing mRNA molecules using poly-T oligo-attached magnetic beads. Three biological replicates were used per condition. After purification, mRNA was fragmented to achieve an average insert size of 200 to 400 bases using divalent cations under elevated temperature. Resulting cleaved RNA fragments were reverse transcribed into first-strand cDNA using reverse transcriptase, actinomycin D, and random hexamer primers. Subsequently, blunt-ended second-strand cDNA was obtained using DNA Polymerase I, RNase H, dUTP, and 2′-deoxyuridine, 5′-triphosphate (dUTP) nucleotides. Adenylation of the resulting cDNA fragments at their 3′ ends was performed followed by ligation with indexing adapters. PCR enrichment was used to subsequently create specific cDNA libraries. The KAPA SYBR FAST ABI Prism Library Quantification Kit (Roche) was used to quantify the libraries. Equimolar amounts of each library were used for cluster generation on the cBot using the Illumina TruSeq PE Cluster Kit v3. Sequencing was carried out on a HiSeq 1000 instrument using the indexed, 50 cycles paired-end read (PR) protocol, and the TruSeq SBS v3 reagents according to the Illumina HiSeq 1000 System User Guide. Bcl files obtained from image analyses and base calling were converted into fastq files using the bcl2fastq v2.18 software (Illumina). Library construction and RNA-seq were performed at the Regensburg University service facility KFB (Competence Center for Fluorescent Bioanalytics).

### Data processing, mapping, differential expression, and statistical analysis

RNA-seq PR quality was assessed using FastQC, followed by trimming and filtering using Trimmomatic v.0.35 (Bolger et al. 2014). Processed PRs were mapped and aligned using the STAR v. 2.5.2 program (Dobin et al. 2013) and the maize reference genome sequence AGPv3 assembly using annotation release-5b+ (corresponding to Gramene AGPv3.27). Alignment results were summarized per gene by identifying read pairs aligned to exonic regions with featureCounts (Liao et al. 2014). Reads overlapping with more than one feature (i.e. gene region) were excluded from the analyses. We performed variance stabilizing transformation of raw counts and analyzed nonmerged technical replicates using R package DESeq2, to evaluate the presence/absence of batch effect and outliers (Love et al. 2014). Differential expression analysis between NS and HS pollen samples was conducted using fold change > 1.5 and *P* < 0.05 (after the false discovery rate adjustment for multiple testing) for the null hypothesis as threshold using DEseq2 (Love et al. 2014).

To elucidate transcriptional correlations, the cor-function was implemented in the R package WGCNA (Langfelder and Horvath 2008) to identify genes with shared expression profile. Genes exhibiting similar expression profiles were considered as seed candidates to obtain direct and indirect interactions using the STRING v11 database (Szklarczyk et al. 2019). A threshold high confidence score of 0.7 was used to construct cluster correlation analysis. Only interactions with high levels of confidence were extracted from the database and considered as valid.

### Flow cytometry analysis

Sperm cells were harvested and used for isolation as described above. Isolated sperm cells were incubated in staining solution (CyStain UV Ploidy, Sysmex) for 5 to 15 min in the dark (Grossman et al. 2021). After staining, samples were further purified using 50 *μ*m filters, and their DNA content was analyzed via flow cytometry using the CyFlow Space system (Sysmex).

### AP-MS analysis of proteins

Mature pollen grains were used for proteomic analyses. For protein extraction, 50 mg of pollen grains were ground with liquid nitrogen using a mortar and dissolved in 250 *μ*L of ice-cold extraction buffer (50 mM Tris/HCl, 150 mM NaCl, 0.1% sodium deoxycholate [*w*/*v*], 0.1% Triton-X100 [*v*/*v*], 1 mM PMSF, pH 8.0). Proteomic analyses were performed as described (Antosz et al. 2020). In brief, total proteins from pollen samples were separated on a 10% SDS–PAGE gel containing the PageRuler Prestained Protein Ladder as a molecular weight marker (Thermo Fisher Scientific). After in-gel digestion with trypsin (Promega), the resulting peptide mixtures were loaded onto the UltiMate 3000 RSLCnano system, which was connected to an Orbitrap Elite hybrid mass spectrometer (MS, Thermo Fisher Scientific). MS data were acquired using a data-dependent strategy that selected the top 10 precursors for higher-energy collisional dissociation (HCD) fragmentation. The analysis of MS raw data files was performed using the Proteome Discoverer software (Thermo Fisher Scientific; version 1.4), utilizing in-house Mascot (Matrix Science; version 2.6) and Sequest search engines. MS/MS ion searches were conducted against the UniProt (The UniProt Consortium et al. 2023) and the Gramene (Tello-Ruiz et al. 2022) protein databases for maize (*Z. mays* L). Postprocessing of search results was performed using Percolator (Spivak et al. 2009). Only peptides with a *q* value < 0.01, rank 1, and a minimum length of 6 amino acids were considered. Protein abundance was determined using the protein area calculated by the Proteome Discoverer software (Thermo Fisher Scientific; version 1.4).

### Statistical analysis

Statistical analyses were performed using R software/environment. For all measurements, data from at least 4 independent experiments were used. Data represented mean and median values including standard deviations. Wilcoxon's singed-rank test was used to compare gene expression between HS and NS plants (Kim et al. 2021). Differences in means were considered significant at *P*-value < 0.05.

### Accession numbers

Ndc80 (Zm00001eb040130), Mis12 (Zm00001eb433940), Mad2 (Zm00001eb425240), BUB3 (Zm00001eb151660), VILLIN3 (Zm00001eb389350), LIM (LIN-11, ISL-1, and MEC-3—Zm00001eb268250), C-CAP (Zm00001eb040800), microtubule-associated protein EB1C (Zm00001eb044540), TBCC (Zm00001eb236410), WAPL (Zm00001eb220920), RCC1 (Zm00001eb049950), and Sister (Zm00001eb412050).

## Supplementary Material

kiae087_Supplementary_Data

## Data Availability

The data underlying this article are available in the article and in its online supplementary material.

## Supplementary data

The following materials are available in the online version of this article.

**Supplementary Figure S1.** Heat stress at the unicellular stage of maize pollen development decreases pollen viability.

**Supplementary Figure S2.** Heat stress during the bicellular stage of pollen development in maize does not affect pollen morphology and viability.

**Supplementary Figure S3.** Gene ontology enrichment analysis of genes differentially expressed in maize pollen in response to heat stress.

**Supplementary Figure S4.** Heat stress alters transcriptional gene expression of highly expressed genes.

**Supplementary Figure S5.** Heat stress at the bicellular stages does not affect DNA content of sperm cells in maize.

**Supplementary Table S1.** List of identified proteins involved in cytoskeletal organization.

**Supplementary Table S2.** Summary of sperm cell samples including number of cells per replicate, aligned pairs mapped unique, and percentage of reads per trimmed reads.

**Supplementary Table S3.** Differentially expressed genes between NS and HS samples during the tetrad stage of pollen development.

**Supplementary Table S4.** List of genes used for gene cluster correlation analysis.

**Supplementary Table S5.** TPM vales of APC/C members under NS and HS conditions.

**Supplementary Table S6.** List of misregulated tubulin-associated genes in sperm cells after heat stress at the bicellular stage.

**Supplementary Table S7.** List of primers used in study.

## Dive Curated Terms

The following phenotypic, genotypic, and functional terms are of significance to the work described in this paper:

SKP1 Gramene: AT1G75950
SKP1 Araport: AT1G75950

pollen AmiGo: PO:0025281

DAPI CHEBI: CHEBI:51231

PGM Gramene: AT1G78050
PGM Araport: AT1G78050

HSP70 Gramene: AT3G12580
HSP70 Araport: AT3G12580
