# Supplementary material for: Heat stress at the bicellular stage inhibits sperm cell development and transport into pollen tubes
Source: Plant Physiol. 2024 Feb 15;195(3):2111–28. doi: 10.1093/plphys/kiae087 (PMC11213256; doi:10.1093/plphys/kiae087)
Supplement: kiae087_Supplementary_Data [file kiae087_supplementary_data.zip › PP2023RA01437R1_Supplemental_Figures.pdf]

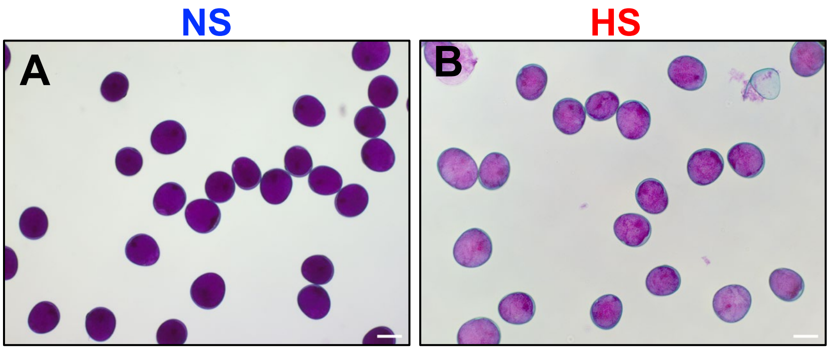

Supplemental Figure S1. Heat stress at the unicellular stage of maize pollen development decreases pollen viability. (A) Light microscopic images of non-stressed (NS) and **(B)** heat-stressed (HS) pollen stained with Alexander staining. Scale bars = 50 µm.

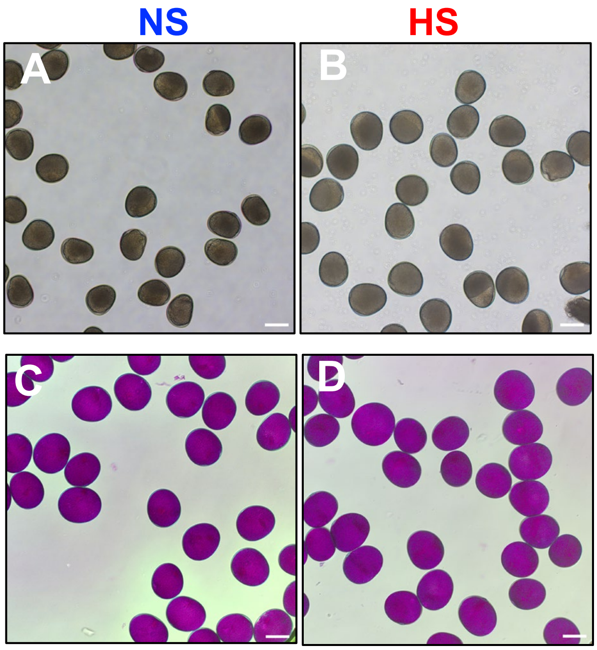

Supplemental Figure S2. Heat stress during the bicellular stage of pollen development in maize does not affect pollen morphology and viability. (A-B) Bright-field images of NS and HS pollen, respectively. **(C-D)** Light microscopic images of non-stressed (NS) and heat-stressed (HS) pollen as indicated after Alexander staining. Differences in pollen viability between both conditions could not be detected. Scale bars = 50 µm.

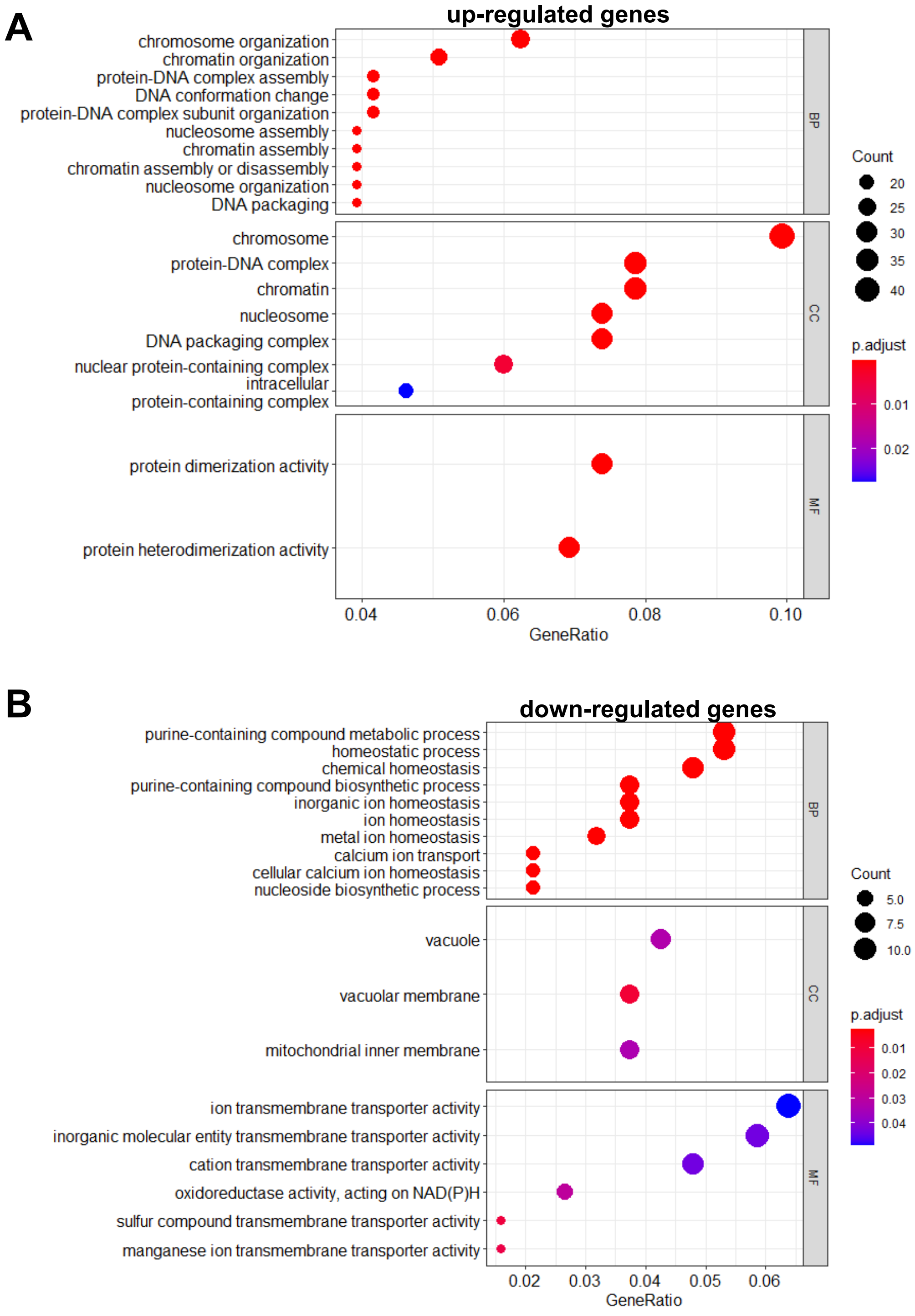

Supplemental Figure S3. Gene ontology enrichment analysis of genes differentially expressed in maize pollen in response to heat stress. (A) Up-regulated and **(B)** down-regulated genes. Differentially expressed genes were categorized in three functional groups: BP: biological process, MF: molecular function, and CC: cellular component. Count numbers and gene ratio (equals the number of differentially expressed genes against the number of genes associated with a GO term in the maize genome) are indicated. Color gradient indicates high enrichment (low p.adjust) in read and blue indicates low enrichment (high p.adjust). Significance threshold was set to adjusted $p<0.05$.

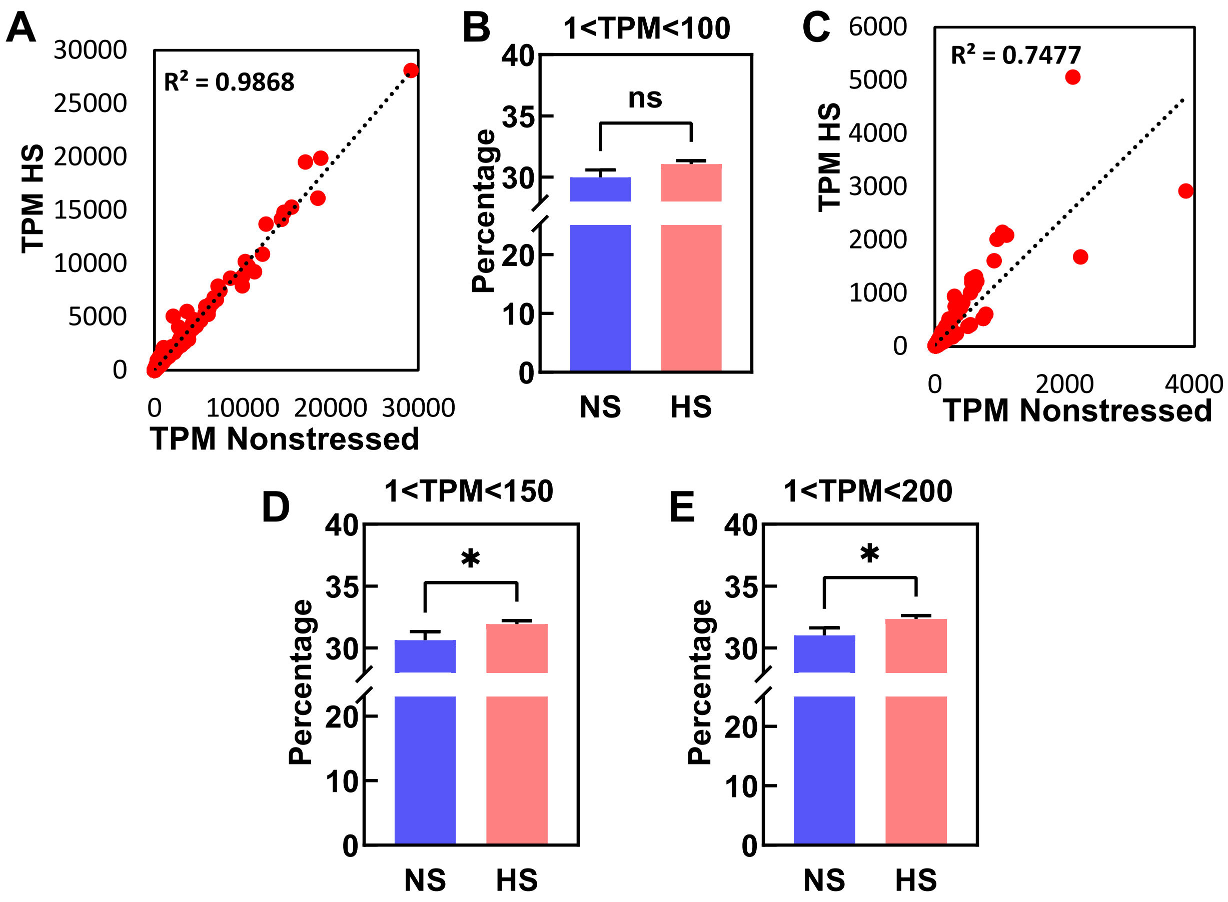

Supplemental Figure S4. Heat stress alters transcriptional gene expression of highly expressed genes. (A) Genome-wide comparison of TMP (transcripts per million) values of sperm cell genes between no-stress (NS) and heat-stress (HS) conditions. **(B)** Percentage of genes expressed in NS and HS sperm cell samples using TPM values >1 but <100. **(C)** Comparison of NS and HS expressed genes with TMP vales >100. (**D** and **E**) Percentage of genes expressed in sperm cells at NS and HS conditions using TPM values >1 but <150 **(D)** and >1 but <200, respectively. Asterisks indicate significant difference at P < 0.01; one-tailed *t*-test was used for comparisons. n = 3 biological replicates. Error bars represent standard deviation.

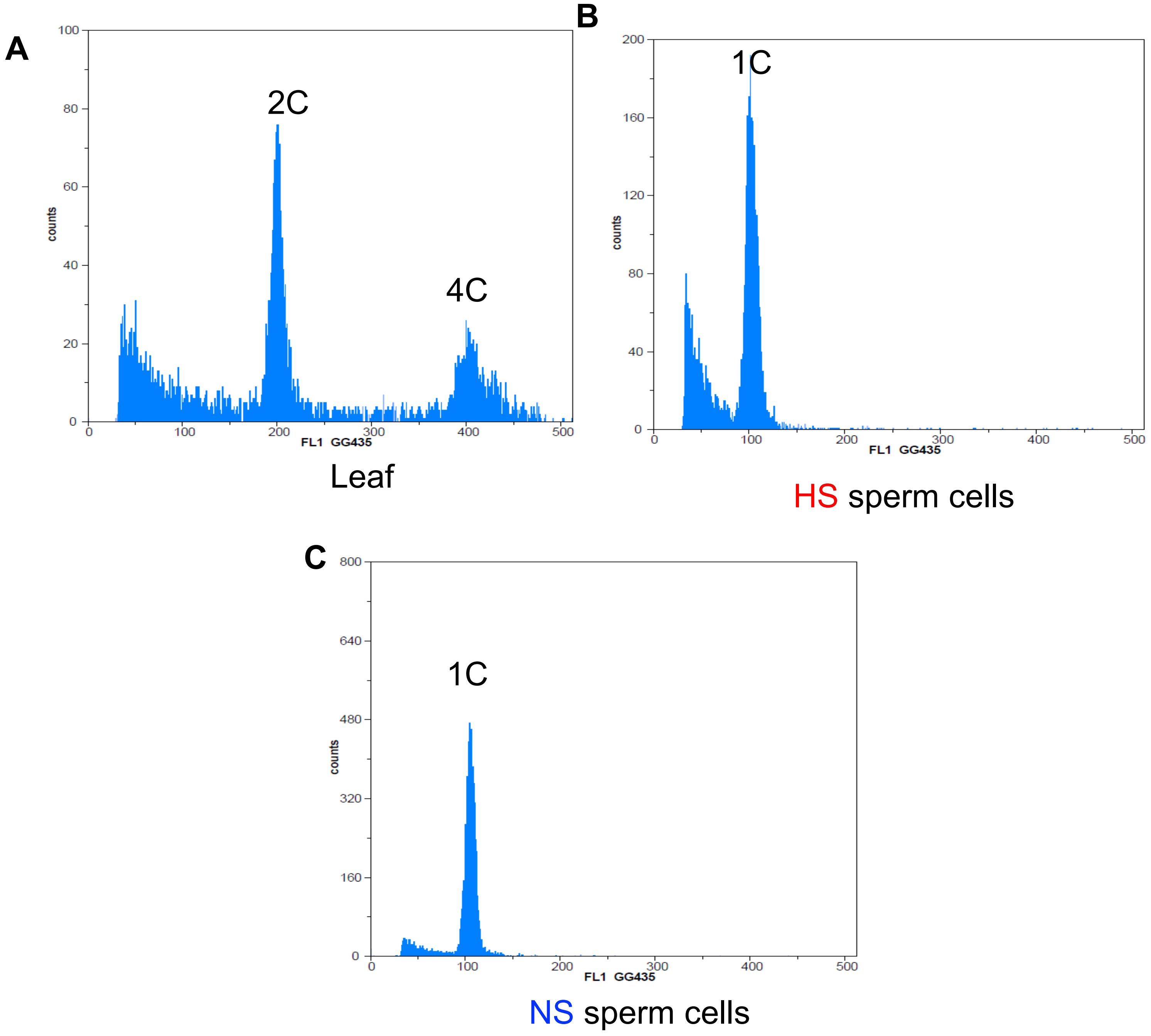

Supplemental Figure S5. Heat stress at the bicellular stages does not affect DNA content of sperm cells in maize. Flow cytometry analysis of **(A)** leaf, **(B)** non-stressed (NS) and **(C)** heat-stressed (HS) sperms cells. The chromatin content (C-values is indicated). While leaf cells appear diploid (2C), sperm cells are haploid (1C) and do not show signatures of aneuploidy.
